# Genetic Diversity, Linkage Disequilibrium and Population Structure of Bulgarian Bread Wheat Assessed by Genome-Wide Distributed SNP Markers: From Old Germplasm to Semi-Dwarf Cultivars

**DOI:** 10.3390/plants10061116

**Published:** 2021-05-31

**Authors:** Vladimir Aleksandrov, Tania Kartseva, Ahmad M. Alqudah, Konstantina Kocheva, Krasimira Tasheva, Andreas Börner, Svetlana Misheva

**Affiliations:** 1 Institute of Plant Physiology and Genetics, Bulgarian Academy of Sciences, Acad. G. Bonchev str., Block 21, 1113 Sofia, Bulgaria; aleksandrov@gbg.bg (V.A.); tania_karceva@abv.bg (T.K.); konstvk@abv.bg (K.K.); krasitasheva@abv.bg (K.T.); 2 Institute of Agricultural and Nutritional Sciences, Martin Luther University Halle-Wittenberg, Betty-Heimann-Straße 3, 06120 Halle (Saale), Germany; ahqudah@gmail.com; 3 Leibniz Institute of Plant Genetics and Crop Plants Research (IPK Gatersleben), Corrensstraße 3, Seeland, 06466 Gatersleben, Germany; boerner@ipk-gatersleben.de

**Keywords:** bread wheat, genetic diversity, linkage disequilibrium, modern cultivars, old germplasm, population structure, SNP markers

## Abstract

Genetic diversity and population structure are key resources for breeding purposes and genetic studies of important agronomic traits in crops. In this study, we described SNP-based genetic diversity, linkage disequilibrium and population structure in a panel of 179 bread wheat advanced cultivars and old accessions from Bulgaria, using an optimized wheat 25K Infinium iSelect array. Out of 19,019 polymorphic SNPs, 17,968 had a known chromosome position on the A (41%), B (42%) and D (11%) genome, and 6% were not assigned to any chromosome. Homoeologous group 4, in particular chromosome 4D, was the least polymorphic. In the total population, the Nei’s gene diversity was within the range 0.1–0.5, and the polymorphism information content ranged from 0.1 to 0.4. Significant differences between the old and modern collections were revealed with respect to the linkage disequilibrium (LD): the average values for LD (*r^2^*), the percentage of the locus pairs in LD and the LD decay were 0.64, 16% and 3.3 for the old germplasm, and 0.43, 30% and 4.1 for the modern releases, respectively. Structure and k-means clustering algorithm divided the panel into three groups. The old accessions formed a distinct subpopulation. The cluster analysis further distinguished the modern releases according to the geographic region and genealogy. Gene exchange was evidenced mainly between the subpopulations of contemporary cultivars. The achieved understanding of the genetic diversity and structure of the Bulgarian wheat population and distinctiveness of the old germplasm could be of interest for breeders developing cultivars with improved characteristics. The obtained knowledge about SNP informativeness and the LD estimation are worthwhile for selecting markers and for considering the composition of a population in association mapping studies of traits of interest.

## 1. Introduction

Bread wheat (*Triticum aestivum*, L.) is an important strategic crop for food security globally. In Bulgaria, bread wheat is the major cereal crop grown on 1.0 to 1.3 million ha land area, with an average annual production of 5.1 million tonnes, and an average grain yield of 4.2 tonnes/ha (data for the last 10 years according to Annual Reports of the Ministry of Agriculture, Food and Forestry). 

The early breeding activities in Bulgaria date back to the beginning of the 20th century when the relatively homogeneous selections made within landraces and, later, crosses involving local and foreign accessions have brought up the first cultivars. In the 1960s, the science-based wheat breeding was launched with the introduction of alleles for reduced height or semi-dwarfism (initially *Rht8*, later *Rht-B1b/d*) and plant adaptability alleles for vernalization response, *Vrn*; photoperiod response, *Ppd*, and frost tolerance, *Fr*) [1,2]. Breeding programs have been focused on improving productivity, grain quality, disease resistance and abiotic stress tolerance, with accent on low-temperature and drought tolerance. Since 1960, more than 140 cultivars have been released in Bulgaria by the two major breeding centres, the Dobrudza Agricultural Institute (DAI) at General Toshevo (Danube Plain, Northern Bulgaria), and the Institute of Plant Genetic Resources (IPGR) at Sadovo (Thracian Lowland, Southern Bulgaria). A small number of cultivars (both modern and old) has been released in research institutes or breeding stations in the Western part of the country. The vast majority of the approved cultivars had been included in the National List and some of them are still in the production system. 

Bread wheat in Bulgaria is cultivated in regions of three climatic environments—continental, transitive-continental and transitive-Mediterranean. In addition to the necessity of yield increase and sustainability for food security, the increasing unpredictability of the weather conditions imposed by climate change during the last 50–60 years [3] entails the development of cultivars of very high productive potential, high adaptability and plasticity to guarantee a sufficient yield under a wide range of environments. Breeders face the task to construct cultivars that are resilient and adaptive to the climate fluctuations, able to withstand early-season drought episodes, inconsistency of winter temperatures and snow cover, water insufficiency and high-temperature stress during flowering and grain filling periods. 

A powerful strategy to respond to these challenges is to explore genetic resources in order to select promising material and incorporate superior traits/genes into new cultivars. Diversity studies of the Bulgarian wheat genepool have been conducted on yield performance and tolerance to water deficiency stress [4], frost tolerance [5], pest resistance [6], nitrogen use efficiency [7], seed storage proteins [8], and leaf morpho-anatomy [9]. Several studies examined the genetic variation at the DNA level using microsatellite markers. These studies surveyed the distribution of *Rht*, *Ppd*, and *Vrn* alleles [1,8,10], and the allelic variation at loci determining frost tolerance [5]. A microsatellite-based comparative analysis within a set of 81 Bulgarian accessions evaluated the current levels and temporal trends of molecular genetic variation, and revealed no declining trends in molecular diversity due to purposeful breeding activities [11]. 

In breeding, there is a constant need for sources of new diversity. While contemporary elite cultivars come from a relatively constricted genetic pool and are mostly designed for high input production systems, landraces and historic cultivars are just such a resource of wide intrinsic genetic variation. Landraces are locally adapted populations with a distinct identity that lack directed improvement, while historic cultivars are considered as relatively homogeneous selections made within landraces, or early breeding releases, that had been cultivated but currently are no longer in production [12,13]. Both types of old germplasm are useful sources of alleles that could play a buffer role in adverse environments, improve crops plasticity and their ability to grow in low-input agro-systems [14,15]. Bulgarian old germplasm is noted for its stable yield, drought tolerance, high protein content, or good bread-making quality [16]. At the DNA level, microsatellite-based studies on this material indicated the existence of high genetic variation and heterogeneity [11,17]. After the 1960s, following the introduction of the semi-dwarf high-yielding cultivars, the cultivation of the landraces and the existing tall cultivars has been abandoned. Seed samples of this generally extinct germplasm are maintained and reproduced at the large European seed gene banks [18]. 

Single nucleotide polymorphisms (SNPs) along with microsatellites are currently the most abundant class of DNA markers and the most valuable genomic tools in plants [19,20]. For model species, but also for crops, SNPs have now become the marker system of choice for a number of applications such as building genetic maps, screening and evaluation of genetic resources, in genome-wide association studies (GWAS), as well as in the marker-assisted selection and genomic breeding [20,21]. 

Characterization of molecular genetic diversity and population structure analysis in the existing Bulgarian bread wheat germplasm have enormous significance for supporting breeding efforts, association genetic studies of important agronomic traits, and resources preservation. For the present study, a panel of 179 accessions, comprising 128 most representative modern semi-dwarf cultivars, 44 historic tall cultivars and 7 landraces has been assembled. The aim was to provide an update of the molecular genetic variation available in the bread wheat genepool in Bulgaria: to characterize the genetic diversity, population structure, and linkage disequilibrium in the collection of contemporary and old accessions of bread wheat using a new optimized 25K SNP genotyping array.

## 2. Results

### 2.1. Genetic Diversity Analysis and SNPs Distribution

**Figure 1:**
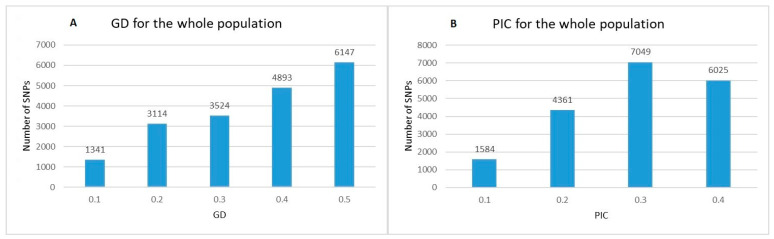
Distribution of Nei’s gene diversity (GD) (**A**) and polymorphism information content (PIC) (**B**) for 19,019 polymorphic SNP markers within a population of 179 old and modern Bulgarian bread wheat accessions.

The analysis of the reduced number of SNPs (19,019) showed a wide range of Nei’s gene diversity (GD) and polymorphism information content (PIC) values across the whole population (Figure 1). The values of GD ranged from 0.1 to 0.5 (Figure 1A). Markers with the lowest GD (0.1) were 1341, while SNPs with GD = 0.5 were maximal in number (6147). The values of PIC ranged from 0.1 to 0.375 (Figure 1B), where the number of markers with PIC = 0.1 was minimal (1584) and the number of markers with PIC = 0.3 was maximal (7049). 

**Figure 2:**
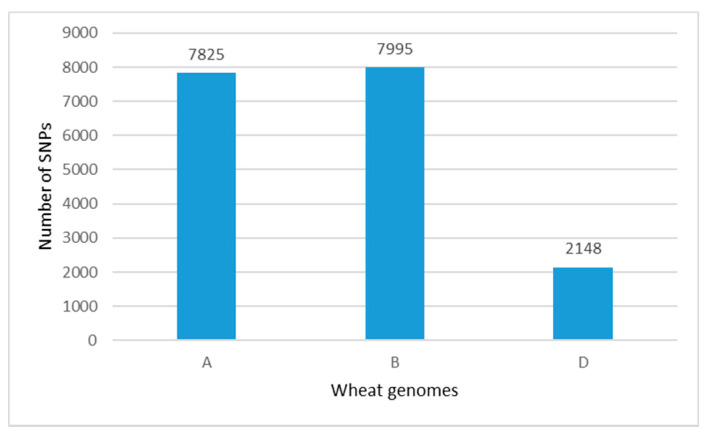
Distribution of SNP markers along the A, B and D genomes within a population of 179 old and modern Bulgarian bread wheat accessions.

The 19,019 SNPs were well distributed across all 21 wheat chromosomes within the set of 179 accessions. A total of 17,968 SNPs had known positions on the A-, B- and D-genome chromosomes (Figure 2), and 1051 SNPs representing 6% of the total number of 19,019 markers were not assigned with any chromosome.

**Figure 3:**
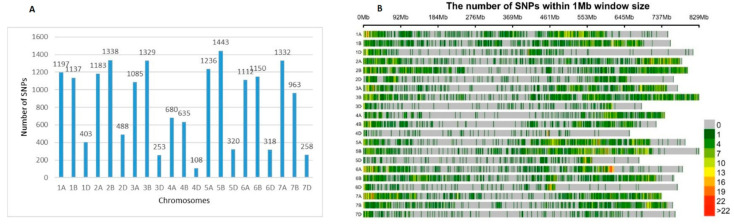
Distribution (**A**) and density (**B**) of SNP markers along the wheat chromosomes within a population of 179 old and modern Bulgarian bread wheat accessions.

The contribution of SNPs number scored on the A and B genomes was similar, 41% and 42%, respectively, while the D genome chromosomes had the least coverage (11%). The number of SNPs ranged from 108 on chromosome 4D to 1443 on chromosome 5B (Figure 3A). Considering the distribution of SNPs across the homoeologous groups, group 4 scored the lowest number of markers (in total 1423, or 7.9% of the total number of markers with a known chromosome position), while the total number of markers on the rest of the homoeologous groups was much higher—from 2553 (group 7) to 3009 (group 2). The number of SNPs within 1 Mbp in each chromosome confirmed that chromosomes in the D genome had the lowest number of SNPs (Figure 3B). 

### 2.2. Differentiation between Old and Modern Germplasm

After the removal of SNP markers with minor allele frequency (MAF) less than 5% as well as those markers with more than 20% missing values, we estimated the SNP number, GD, PIC and linkage disequilibrium (LD) patterns for each chromosome of the 128 modern cultivars and the 51 old ones (Table 1, Table S1).

From the total number of 19,019 markers, 15,289 (80%) were polymorphic in both collections, 2077 (11%) markers were polymorphic only in the group of old accessions, while 1653 (9%) were polymorphic only in the modern cultivars. The number of polymorphic markers with a known chromosome position was 16,942 in the set of old accessions, while in the modern collection this number was higher (17,366). From the total of 17,968 polymorphic markers with a known chromosome position, 1026 (6%) markers were polymorphic only in the old accessions, 602 (3%) only in the modern cultivars, and 16,340 (91%) were polymorphic in both collections. In both sets of accessions, the D genome was characterized with the lowest SNP number, while A and B genomes had much higher coverage (Table 1, Figure 3B), more evident within the modern collection. Within the old group of accessions, GD ranged from 0.33 (chromosome 4D) to 0.4 (1A) with an average value of 0.36, and PIC ranged from 0.27 (chromosome 4D) to 0.31 (1A, 1D) with an average value of 0.29. For the modern cultivars, GD ranged from 0.32 (chromosome 5D) to 0.39 (1D) with an average value of 0.35, and PIC ranged from 0.27 (chromosomes 1B, 4A, 5D, 6A) to 0.31 (1D) with an average value of 0.28. The differences between the two groups of accessions regarding GD were slight but statistically significant for 11 chromosomes, the A, B and D genome, and for the average estimation. Regarding PIC, the differences between the two collections were significant for 10 chromosomes, the A and B genome, and for the average value (Table 1, Table S1).

### 2.3. Linkage Disequilibrium

For both sets of accessions, linkage disequilibrium (LD) characteristics (square of marker correlations r^2^, the percentage of locus pairs having significant LD at *p* < 0.001, and LD decay) were estimated for individual chromosomes and genomes (Table 1, Table S1). 

In the group of old cultivars, LD (*r^2^*) ranged from 0.45 (chromosome 7D) to 0.71 (2D, 3D, 4B, 6D, 7A) with an average of 0.64, while in the modern set, LD (*r^2^*) ranged from 0.34 (chromosome 4D) to 0.56 (chromosome 6D) with an average of 0.43. Both selections (old and modern) had lowest LD (*r^2^*) in the B genome (0.63 and 0.40, respectively). The old cultivars had highest value of LD (*r^2^*) in the A genome—0.65, while the modern cultivars had highest LD (*r^2^*) value in the D genome—0.47. LD (*r^2^*) mean values for each chromosome and genome, and the average one were significantly higher in the old germplasm.

In the old collection, the percentage of locus pairs having significant LD at *p* < 0.001 ranged from 6% (chromosome 4A) to 27% (chromosome 6A) with an average value of 16%, while in the modern collection this parameter ranged from 10% (chromosome 7D) to 45% (chromosome 1B) with an average value of 30%. The lowest percentage of markers with significant LD at *p* < 0.001 was in the D genome—12% and 21% for the old and modern sets, respectively. The old cultivars had highest percentage of locus pairs with significant LD at *p* < 0.001 in the A genome (19%), while in the group of modern ones this statistic had highest value in the B genome—39% (Table 1). 

In the old germplasm, the values of LD decay ranged from 1 (chromosomes 6A, 6D) to 6 cM (chromosomes 1A, 4B, 4D). In the modern collection, LD decay ranged from 2 (chromosomes 2A, 6A) to 8 cM (chromosomes 2D, 4D). On average, the LD decay was higher in the modern collection (4.1) compared to the LD decay value (3.3) in the old germplasm. 

### 2.4. Population Structure

**Figure 4:**
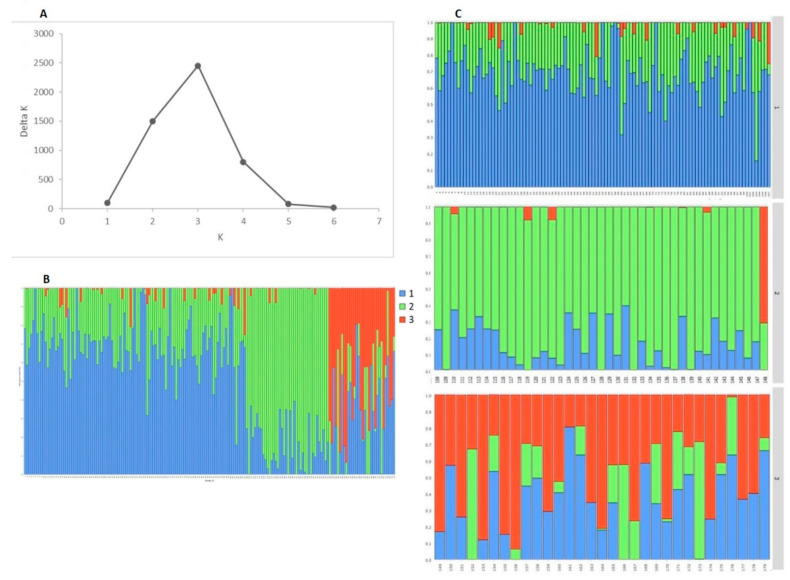
Genetic structure of Bulgarian old and modern bread wheat accessions. (**A**) Estimation of the optimal number of clusters, defined by the Evanno method. (**B**) Inferred structure of a panel of 179 accessions. Each individual is represented by a colored bar with a length proportional to the estimated membership to each of the three clusters. (**C**) Plots in right depict the clusters as separate subpopulations (SPs): SP1 (109 accessions); SP2 (49 accessions); SP3 (16 accessions) and 5 were admixed.

Following the Evanno method in STRUCTURE Harvester software, the optimal number of subpopulations (SP) (ΔK) was 3 (Figure 4A). The three subpopulations (SP1, SP2 and SP3) are depicted in Figure 4B,C. According to the STRUCTURE software results, 103 accessions out of 179 showed a strong membership coefficient (Q-value) to one of the SPs (Q > 0.7), 76 varieties had moderate Q value to one of the SPs (0.5 < Q < 0.7), and five cultivars were admixed with low Q value (Q < 0.5) to one of the SPs. The largest subpopulation (SP1, Qmean = 0.708) comprising 109 accessions included predominantly modern releases (103 accessions), most of them developed in the two major breeding centres in the Northern and Southern part of the country, and six old accessions (Table S2, Table 2). The second-largest subpopulation (SP2; Qmean = 0.798) included accessions belonging almost exclusively to the old germplasm, 43 out of 49, most of them coming from Northern Bulgaria, and six modern cultivars (Table S2, Table 2). The smallest cluster, SP3 (16 accessions; Qmean = 0.724) contained also mostly modern cultivars (14) from the Northern and Southern breeding regions, and two old accessions.

### 2.5. Cluster Analysis

**Figure 5:**
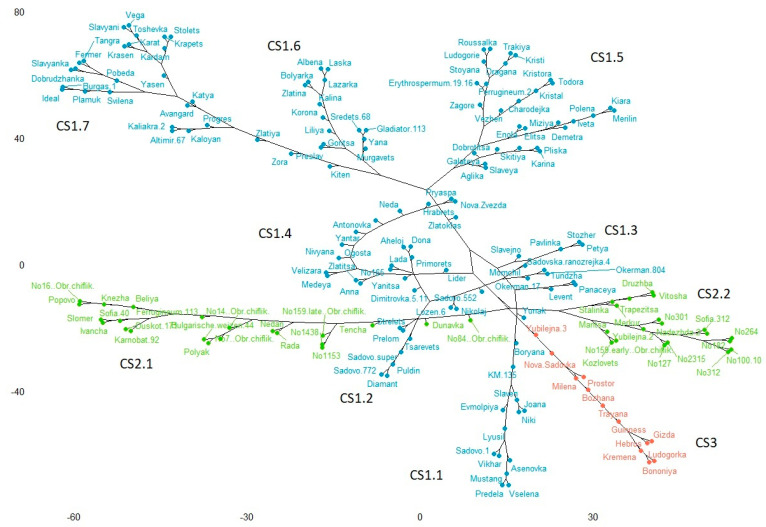
A neighbor-joining dendrogram is inferred by the k-means clustering algorithm. Correspondence with SPs, obtained by STRUCTURE: the largest cluster (blue) corresponds to SP1; the green cluster corresponds to SP2 and the red cluster corresponds to SP3.

The k-means clustering algorithm was applied using the common SNP markers between the old and modern accessions. This approach successfully discriminated the two germplasm types. The resulting neighbor-joining dendrogram (Figure 5) consisted of three clusters (CS), which were largely in agreement with the results obtained by STRUCTURE (Figure 4). The largest cluster in the neighbor-joining tree designated as CS1 (125 entries, in blue) corresponds to SP1, including predominantly modern releases and only five old accessions. This cluster includes all 109 SP1 accessions plus 16 entries belonging to SP2 (eight), SP3 (three) and the five admixed cultivars. In this cluster, we defined seven branches. Branch CS1.1 contained 14 modern cultivars, of which 13 originated from the Southern breeding centres and only one (Asenovka) was coming from the Northern region; two out of these 14 entries were admixed (KM-135 and Evmolpiya). Branch CS1.2 had 19 entries distributed in three subbranches, including 14 modern and five old accessions; the majority was originated from the Southern region (15), three accessions came from the Northern region (Dona, Dimitrovka 5-11 and No165) and one from the Western region (Lozen-6).

Twelve of the accessions in branch CS1.2 corresponded to the STRUCTURE SP2, 6 to SP1 and 1 (Sadovo 552) was classified as admixed. Branch CS1.3 was the most diverse one, including eight modern cultivars; all of them but one (Levent) coming from Southern Bulgaria. These eight accessions belonged to all STRUCTURE subpopulations: SP1 (4), SP3 (2), SP2 (1), and one (Pavlinka) was classified as admixed. Branch CS1.4 had 14 members, all of them from the modern collection belonging to the STRUCTURE SP1. All of them were from Northern Bulgaria, except for two (Hrabrets and Nova Zvezda), coming from the Southern breeding centres. All 29 accessions placed in branch CS1.5 were classified by STRUCTURE as SP1, the majority (27) being modern releases, and only two were old accessions (Erythrospermum 19-16 and Ferrugineum-2), all of them originated from the Northern breeding region. Branch CS1.6 had 14 modern members classified as SP1 from the STRUCTURE software. Most of them (10) originated from the Northern breeding region, two (Murgavets and Yana) were releases of the Southern breeding centre and other two (Gladiator-113 and Sredets-68) were created by the Western breeding institute. The last branch CS1.7 contained 27 entries revealed as SP1 according to STRUCTURE, 23 of them being modern and four old accessions (Burgas-1, Kaliakra-2, Plamuk and Dobrudvanka). Cultivar Kiten in this branch was classified by STRUCTURE as admixed; seven accessions were from Southern breeding centres, one (Altimir-67) was from Western Bulgaria, and the remaining 19 were coming from the Northern region. 

The second-largest cluster CS2 (41 entries, in green) corresponded to SP2, including exclusively the old cultivars and landraces, and only three modern releases. This cluster can be divided into two branches: CS2.1, consisting of 22 entries, of which 15 from the Northern region, five from the Southern and two from the Western part of the country. In this branch, the cluster analysis placed one modern cultivar (Rada). Branch CS2.2 comprised 19 accessions, of which two (Druzhba and Trapezitsa) were modern ones. The distribution according to the geographic region was: Northern—11, Southern—six and Western—two. The smallest cluster CS3 (13, in red) corresponded entirely to SP3. Ten entries in this cluster were contemporary ones and only two (Nova Sadovka and Yubilejna-3) belonged to the old germplasm. 

**Figure 6:**
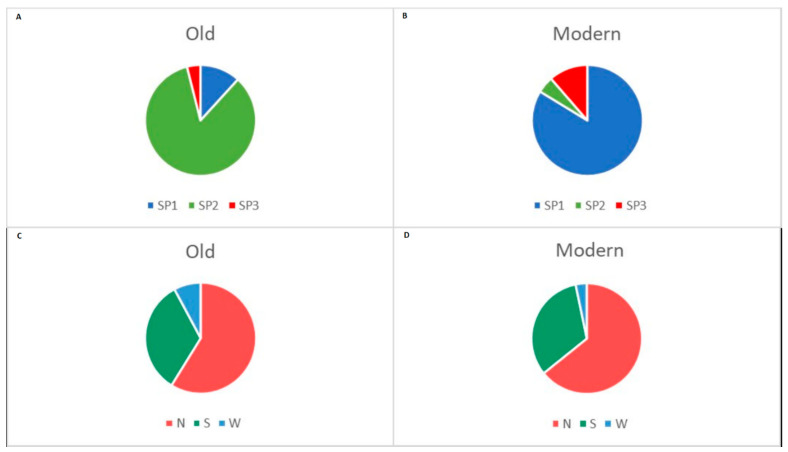
Distribution of old (**A**) and modern (**B**) accessions depending on their assignment to the STRUCTURE subpopulations (SPs). Distribution of old (**C**) and modern (**D**) accessions according to the geographic origin—breeding centre/collection site (N, Northern; S, Southern; W, Western part of Bulgaria).

Most of the members of the old group (84%) fell into SP2, 12% belonged to SP1 and only two cultivars representing 4% were placed in SP3 (Figure 6A). The group of modern semi-dwarf cultivars again was unequally distributed between the three subpopulations: 84% of them belonged to SP1, 11% to SP3 and only 5% to SP2 (Figure 6B). Fifty-nine percent of the old accessions came from the Northern region of the country, 33 and 8% originated from the Southern and Western parts, respectively (Figure 6C). Seventy-nine members of the modern group (64%) were selected in the Northern breeding region, 40 members (33%) were released in the Southern breeding centres, and only four cultivars (3%) came from the Western research institute with breeding activities (Figure 6D). 

**Figure 7:**
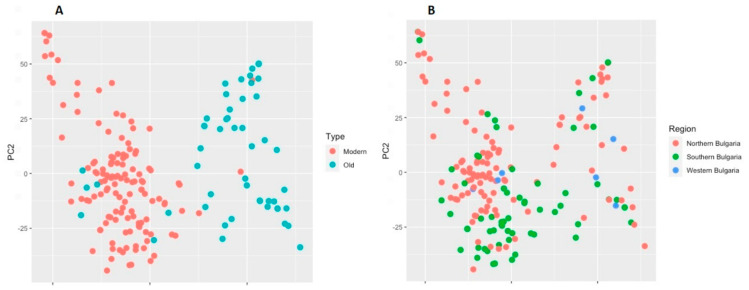
Principal component analysis (PCA) of 179 Bulgarian bread wheat accessions. (**A**) Differentiation between old and modern accessions; (**B**) Differentiation between accessions according to the region of breeding or collection site.

The relationship between the old germplasm and modern releases was also analyzed by principal component analysis (PCA) as a complementary approach to illustrate the clustering mode. In compliance with the results obtained with STRUCTURE and cluster analysis, the first two components of the PCA clearly distinguished the old cultivars from the modern ones (Figure 7A). The two principal components explained 12.2% of the total variation (PC1 8.6% and PC2 3.6%). Applying PCA, we were also partly able to differentiate the accessions according to the region of breeding or collection site (Figure 7B). The cultivars selected in Northern and Southern breeding centres were distributed relatively well in two major groups with a certain admixture portion, while cultivars that originated from the Western region were distributed randomly.

The total genetic diversity *H_T_* of the 179 Bulwheat accessions was 0.3586 and ranged among the three subpopulations (SPs) from 0.3289 for SP3 to 0.3419 for SP2 (Table 3). The values of the diversity estimators: total genetic diversity *H_T_*, mean diversity within each subpopulation *H_S_*, Jost’s index of population differentiation *D_ST_*, and the coefficient of genetic differentiation *G_ST_* were slightly higher for the old cultivars compared to the corresponding values for the modern collection. This agrees with the higher *H_T_* value for SP2, which includes the major part of the old germplasm, compared to SP1 and SP3. The modern cultivars, however, showed a higher value for the gene flow estimator *Nm* (3.08), suggesting higher gene exchange between the modern subpopulations, SP1 and SP3. 

The population differentiation (*D_ST_*) between SPs was low (0.0242) and this led to a low mean value of the coefficient of genetic differentiation *G_ST_* (0.0675) among the SPs (Table 3). As *G_ST_* is defined as the proportion of genetic diversity that resides among subpopulations, this value of *G_ST_* gives information that about 7% of the total genetic diversity is among subpopulations, the remaining 93% representing variation within subpopulations, and that generally the genetic differentiation between the SPs is low. The total value of gene flow *Nm* for SPs (6.91) indicates that there is a certain level of gene exchange between the subpopulations. Comparison between the SPs showed that both statistics *D_ST_* and *G_ST_* had similar values in the pairs SP1-SP2 and SP2-SP3, while *D_ST_* and *G_ST_* values in the pair SP1-SP3 were considerably lower (0.0128 and 0.0380, respectively). Correspondingly, the gene flow estimator *Nm* had highest value in the pair SP1-SP3 (12.66), whereas the pairs SP1-SP2 and SP2-SP3 had significantly lower *Nm* values (Table 3).

## 3. Discussion

### 3.1. Genetic Diversity and SNPs Distribution

Genetic diversity is a main source of biodiversity, which represents any measure that quantifies the magnitude of genetic variability within a population [22]. In bread wheat, two major diversity bottleneck events are recognized. The first one occurred ca. 8000 years ago and led to the emergence of hexaploid wheat [23]. The second bottleneck occurred in the middle of the last century when semi-dwarfing (*Rht*) genes were introduced into then high-productive cultivars thus resulting in a grain yield outbreak known as the Green Revolution. However, the practice of modern intense breeding was suggested to cause inevitably a reduction in the genetic variation available for crop improvement [24]. The concept of ‘genetic erosion’ first presumed by Harlan [25] discusses the possible dramatic shift in population structure or allele frequencies within a species because of purposeful breeding activities. Since then, numerous studies have been devoted to investigate the extent and temporal trends in genetic diversity and the possibility to safeguard natural genetic variability and genomic integrity. Knowledge about genetic diversity in a crop population is fundamental for dealing with multiple biotic and abiotic stress factors that limit crop productivity and, therefore, is imperative to secure food for the human. Genetic variation becomes also an important tool for breeding purposes providing a wider choice of superior parents carrying novel alleles. Especially, the diversity of old germplasm needs characterization and evaluation to incorporate its yet underutilized potential for breeding purposes.

In this study, we described aspects of DNA genetic diversity, population structure and linkage disequilibrium using the currently adopted markers of choice (SNPs) in a bread wheat panel comprising both old accessions and contemporary cultivars from Bulgaria. Nei’s gene diversity (GD) and polymorphism information content (PIC) are important guidance for breeding programs. Genetic diversity at DNA level is a reflection of similarities and differences in the genes of individuals. The gene diversity estimator GD lies between 0 (no differentiation in the population) and 1; higher GD values are associated with higher population differentiation. In our current study, the GD values ranged from 0.1 to 0.5. The PIC, a statistic used to measure the informativeness of a genetic marker for linkage studies varies between 0 (one allele at the marker locus) and 1 (infinite number of alleles); a gene or marker with only two alleles has a maximum PIC = 0.375, and PIC value 0.44 is considered to be moderately informative [26]. In this study, we identified 5944 low informative and 13,075 moderately informative SNP markers according to the Botstein classification [26] (Figure 1). Evaluating SNP genetic diversity demonstrated, in general, lower scores for both PIC and GD compared to outcomes based on SSR (simple-sequence repeats) markers. Thus, Landjeva et al. [11] studied a portion of the current Bulwheat panel, consisting of 77 modern and 14 old cultivars (in total 91) using 19 wheat SSRs and one secalin-specific marker for rye chromosome arm 1RS. The PIC values ranged from 0.1 to 0.82 (average 0.51). The average GD was 0.64 for the old germplasm and 0.61 for the group of modern releases. Previous studies using multiallelic markers such as microsatellites (SSRs) also reported higher levels of polymorphism for both bread and durum wheat [27,28,29,30]. 

However, the obtained results in the current study are in agreement with findings for other wheat populations. In a study of 250 Nebraska winter wheat accessions with GBS-derived SNPs, Elather et al. [31] reported the same values for gene diversity (range from 0.1 to 0.5, average 0.3) and PIC (range from 0.1 to 0.4, average 0.25). The study of Alipour et al. [32] on 369 Iranian wheat accessions, however reported a different range for both GD (from 0.144 to 0.200, average 0.179) and PIC (from 0.003 to 0.375, average 0.172). 

The interest of scientists towards the old germplasm (landraces and old historic cultivars) has been driven by its intrinsic wide genetic variability thus being an important genetic resource for enhancing modern crops with new alleles. This nearly extinct germplasm could play a buffer role in case of adverse environmental conditions and needs characterization and evaluation [14,24]. Our earlier SSR-based study on Bulgarian germplasm [11] indicated high levels of genetic diversity and heterogeneity within the old accessions. The high genetic diversity values characteristic for the old germplasm have been maintained unchanged by the wheat breeders after the 1960s and there seemed to have been a shift of dominating alleles from the old cultivars to those released later, loss of some alleles but the introduction of novel ones. In the present SNP-based study, we compared the levels of diversity between the two collections (old and modern cultivars), and we found significant though slight differences, both GD and PIC having higher scores within the old accessions (Table 1). Within each set of accessions, the A, B, and D genomes were characterized by similar GD values, with a trend of slightly higher scores for the D genome (Table 1). The PIC value for the D genome was equal to those for A and B genomes in the old germplasm, while in the group of modern releases the PIC value for the D genome was slightly higher compared to those for the other two genomes. Other authors [33], in their SNP-based study of genetic diversity within a panel of 354 Mediterranean landraces and improved bread wheat cultivars also reported similar total scores of gene diversity and PIC for both old and modern germplasm. However, in the collection of landraces, the D genome was characterized by definitely lower scores for both gene diversity and PIC compared to the A and B genomes [33]. At the same time, in the group of the advanced cultivars, the D genome showed a slightly higher value for gene diversity than the A and B ones. A study of Iranian bread wheat landraces and cultivars [32] suggested high differentiation not only between landraces and modern cultivars but also between landraces, based on various diversity statistics. Thus, the average expected heterozygosity *He* (Nei’s gene diversity, GD) differed significantly among the contemporary cultivars and the two groups of landraces. A very recent study in durum wheat from Ethiopia by Alemu et al. [34] using high-density SNP markers also differentiated the collections of landraces and modern cultivars based on genetic diversity parameters. Thus, modern cultivars scored higher values of Nei’s gene diversity (0.297) and PIC (0.240) compared to the landrace collection (0.213 and 0.173, respectively). The depicted trends in the differences between modern and old germplasm regarding diversity statistics could reflect a number of factors, among which the proximity of the region to the centre of cereal diversity, geographic region and environmental conditions during the collection of samples, seed storage conditions, or other factors affecting the *per se* genetic variability of the old germplasm. The observed differences in the genetic diversity between old and modern collections could be also attributed to some anthropogenic factors such as seed exchange, and use of various parental sources in breeding programs. 

In the current study, we also observed strong differences in terms of proportion of SNPs with known chromosome assignment between genomes and homoeologous groups. The D genome was the least polymorphic (11%), while A and B genomes displayed almost equal coverage (41 and 42%, respectively), and group 4 chromosomes had the smallest number of SNPs (7.9%) of those with known chromosome location. This observation completely agrees with the outcome of the whole hexaploid wheat genome resequencing from a number of European, Australian, Chinese and Israeli accessions [35]. They also found that the D genome was the least covered with SNPs (10%), while B genome was more polymorphic (49%), followed by the A genome (41%). Similarly, to our findings, they also reported that the SNP density on homoeologous group 4 was the lowest, representing only 8% of the whole SNPs. The observation that the B genome has the highest number of polymorphic markers, and the D genome is the least polymorphic has been reported in other studies on various wheat panels as well [32,36,37]. Our result on the least number of SNPs on chromosome 4D (0.6% of all markers with a known chromosome position) supports a number of earlier studies on various populations using a different number of SNPs [32,36,37]. 

The outcome of our study about the least D genome polymorphism extent is also in lineage with a comparative study between elite bread wheat cultivars and synthetic hexaploid wheats (developed by artificial hybridization between tetraploid wheat, *Triticum turgidum*, with the D-genome progenitor, the diploid wild goat grass *Aegilops tauschii*) using nearly 36,000 high-quality SNPs [38]. According to the outcome of the above study, the D genome polymorphism was generally much lower than the A and B genomes in the elite cultivars, while in synthetic wheats the proportion of SNPs was almost equal between the three genomes. The time of evolution of a genome is supposed to affect the SNP density on chromosomes [32,39].

### 3.2. Linkage Disequilibrium and Population Structure

Linkage disequilibrium (LD) is the nonrandom association of alleles at different loci that reflects their proximity and correspondingly, the probability of recombination breaking the haplotype on which they are found [40]. LD is a sensitive indicator of the population genetic forces that structure the genome, and can be affected by various factors [41]. In particular, LD is strongly influenced by the classification of accessions into groups and unequal distribution of alleles within different groups, which can result in false associations [33]. LD rapidly decays with genetic distance and so it is important to determine the LD patterns, the extent of LD and LD decay to reduce spurious marker-trait associations in further association mapping analyses using this population [41]. In our study, the average values for the square of marker correlations LD (*r^2^*) were significantly different between the old and modern collections. The LD (*r^2^*), the percentage of the locus pairs in LD calculated for intra-chromosomal loci and LD decay were 0.64, 16% and 3.3 for the old germplasm, and 0.43, 30% and 4.1 for the modern releases, respectively (Table 1). The higher LD decay within genomes B and D in the group of modern cultivars probably accounts for the higher average LD decay. This information is valuable in considering the composition of a population and selecting the markers for further association mapping studies of agronomically important traits. The elevated level of LD in crop populations suggests that a smaller number of markers can provide sufficient genome coverage for finding marker-trait associations [41]. In our study, the variation in the LD extent between the old and modern groups suggests that natural selection and purposeful breeding for specific alleles had a different impact within the two sets. The lack of significant difference in the extent of LD in the three genomes in both old and modern collections mirrors the lack of recent introductions from the D genome progenitor and diversity bottleneck event associated with the origin of hexaploid wheat. This observation might reflect the more distant location of Bulgaria from the centre of diversity. 

Analysis of population structure is worthwhile for understanding genetic diversity and for association mapping studies. The Bulwheat old and contemporary accessions were allocated to three subgroups, which was confirmed by the two applied approaches—STRUCTURE and k-means clustering algorithm. Not surprisingly, the vast majority of the old germplasm (landraces and historic cultivars) formed a distinct group using both methods. The outcome of the PCA also supports the results obtained by STRUCTURE and the cluster analysis with respect to the differentiation between the modern and old collections (Figure 7A). However, there was no good distinction between the accessions depending on the geographical origin as suggested by the PCA (Figure 7B) and by the STRUCTURE outcome (Table 2). Table 2 and Figure 6 show that the number of accessions in the Bulwheat population originated from the Northern breeding region is almost twice the number of those coming from the Southern part of the country, and this ratio applies for both modern and old accessions. This distribution was well evident in the STRUCTURE-derived subpopulations SP1 and SP2 (Table 2). The cluster analysis did not discriminate the old accessions in relation to the breeding/collection regions; however, the results by this analysis demonstrated more clear discrimination of the modern cultivars with respect to the breeding region. Thus, branches CS1.1, CS1.2 and CS1.3 of the largest cluster consisted almost exclusively of accessions from the South, while branches CS1.4 to CS1.7 included predominantly accessions that were developed in the Northern region of the country. The presence of a structure of the Bulgarian wheat population could be explained by several factors. First, pedigree evaluation of the modern cultivars, which formed subpopulations SP1 and SP3, inferred by STRUCTURE, and cluster CS1 revealed by the k-means cluster analysis, indicated that some lines appeared frequently in their genealogy (Table S2). This can be supported by at least several examples. For instance, cultivars Nivyana, Velizara, Medeya, and Anna have cultivar Yantar (=Avrora x Era) as a parent and form a close group (branch CS1.4, Figure 5). Closely related are cultivars Kristal, Todora and Kristora (branch CS1.5, Figure 5) all having the Russian cultivar Avrora in their genealogy. A key cultivar in the breeding practice in Bulgaria is Pliska, having cultivar Roussalka as a parent, which in turn has been released in 1971 by a cross with a local accession. These two cultivars had been widely used by the breeders and this is one of the reasons for the observed close relationships between some entries: between Roussalka, Trakya, Ludogorie and Stoyana (branch CS1.5); between Pliska, Karina, Slaveya, Aglika and Galateya (branch CS1.5, Figure 5). In the same branch, cultivars Dragana, Kristi and Trakya, are closely related since Trakya is ancestor in the pedigree of the two former ones (Table S2). In branch CS1.6 in the dendrogram, cultivars Sredets-68, Murgavets and Gladiator-113 are closely grouped, based on their common ancestors Skorospelka-35 and Mexipac (Figure 5, Table S2). In the same branch, close genetic similarities are observed among cultivars Albena, Laska, Lazarka, Bolyarka and Korona, all of them having Romanian lines in their genealogy (Figure 5, Table S2). Considering the period of cultivar release, a temporal trend could be noticed. Initially, the breeders used predominantly Russian, Italian and Serbian cultivars. Further cultivars developed in the 1980s were mostly based on the already created own breeding material, but after the 1990s new and more diverse accessions were introduced in the breeding programs, namely resource from the USA, Romania, Ukraine, France, and CIMMYT breeding material [42]. Experimental mutagenesis and, to some extent, double-haploid approach and wide crosses have been exploited for breeding purposes, as well. Second, traditional breeding for high productivity was associated with selection of favorable traits, which had resulted in the introduction of genes conferring semi-dwarfism, photoperiod-insensitivity, disease resistance, low-temperature tolerance, drought resistance and earliness, and good grain quality [1,5,10,11,43]. Third, the formation of genetically differentiated groups of modern cultivars released by the main breeding centres of different geographic locations might be influenced by the environment-driven selection. The specific agro-ecological conditions where the breeding occurs affects the introduction and maintenance of particular alleles [11], but might also affect the extent of mutation events thus resulting in the polymorphisms at the DNA level.

### 3.3. Population Differentiation

Genetic structure within populations and gene flow between them provide information about the population differentiation. The gene flow is the transfer of genetic material from one population to another. If the rate of gene flow between two populations is high (evidenced by high *Nm* values) then the differentiation of the populations is low and they are considered as a single effective population [44]. In our study, the set of modern cultivars had lower value for the genetic differentiation estimator *G_ST_* and, respectively, higher value for the gene flow estimator *Nm* compared to the corresponding values in the old collection, which is suggestive for higher levels of gene migration within the set of modern releases. These results are in agreement with the population structure and the neighbor-joining dendrogram. The STRUCTURE-derived subpopulation SP2 and the corresponding cluster CS2 in the dendrogram included mostly historic cultivars and landraces. The known information about the pedigree of some old accessions from SP2 (e.g., Nadezhda-2, Okerman-17, Okerman-804, Yubilejna-2, Beliya, No159, and Karnobatska ranozrejka) suggests some extent of gene exchange between the representatives of the old germplasm. Subpopulations SP1 and SP3 and the corresponding clusters CS1 and CS3 consisted mainly of modern releases and a few old accessions. The existing high gene exchange between these two groups is evidenced by low value of the genetic differentiation estimator *G_ST_* (0.0380) and high value of the gene flow statistic *Nm* (12.66, Table 3) in the pair SP1-SP3. This assumption is also highly supported by the genealogy information provided in Table S2. The higher *G_ST_* values and lower *Nm* values obtained for the subpopulation pairs involving old accessions (SP1-SP2 and SP2-SP3, Table 3) signs for a low utilization of the old germplasm by the breeders. For example, the SP3 modern cultivars Hebros, Kremena and Zlatoklas had been derived from crosses with old cultivars. Some SP1 advanced cultivars, for example, Roussalka, Katya, Sadovo 1, Zora, Pliska, Trakya, Yasen, Kaloyan, and Lider had a landrace or other old accession either as a direct parent, or as a more distant ancestor. 

## 4. Materials and Methods

### 4.1. Plant Material

The plant material consisted of a germplasm collection of 179 bread wheat (*Triticum aestivum* L.) accessions originated from Bulgaria (Bulwheat population). The Bulwheat population included 128 modern semi-dwarf cultivars and 51 representatives of the old germplasm of tall stature (Table S2). Seven accessions of the old germplasm corresponded by definition to landraces or selections from landraces from different regions of Bulgaria, and 44 entries were releases of early purposeful breeding based on crosses between local and foreign accessions before the era of semi-dwarfism. Old germplasm was provided by the seed gene banks at the Leibniz Institute for Plant Genetics and Crop Research (IPK), Gatersleben, Germany, and RICP (Research Institute of Crop Production, now Crop Research Institute), Prague, Czech Republic. Seeds from the modern cultivars were provided by the national seed gene bank at the IPGR, Sadovo, the DAI breeding centre, as well as by breeders. 

### 4.2. SNP Genotyping

DNA extraction and SNP genotyping of the Bulwheat panel was performed by SGS Institut Fresenius GmbH TraitGenetics Section (Gatersleben, Germany). 

For genomic DNA extraction, a bulk sample of leaf tissue was taken from ten young seedlings for each accession. The DNA extraction was performed using an in-house CTAB-based protocol with chloroform extraction and isopropanol precipitation, followed by ethanol washing and resuspending the dried pellet in pure water. To generate SNPs, an optimized wheat 25K Infinium iSelect array was used. The array contains 24,145 SNPs combining 17,229 markers from the Illumina 20K array, 6916 new markers from the 135K Axiom array, and additional trait- and gene-specific markers. SNP discovery was performed with the GenomeStudio Project software package (Illumina, San Diego, CA, USA). The reference genome v1.0 of the Chinese Spring genome assembly from the International Wheat Genome Sequencing Consortium (IWGSC_WGA_v1.0) [45] and the genetic map based on the International Triticeae Mapping Initiative (ITMI) Double Haploid population [46] were used for SNP chromosome alignment. In order to reduce possible errors in the performed analyses, the identified SNPs with minor allele frequency (MAF) less than 5% and markers with more than 20% missing values were removed from the dataset. After applying these filters, the reduced number of polymorphic SNPs across the population of 179 accessions was 19,019. SNP density was determined within 1 Mbp in each chromosome according to the recent approach by Yin et al. [47].

### 4.3. Genetic Diversity Analysis

The MAF and polymorphism information content (PIC) were deliberated using PowerMarker software V 3.25 [48]. PIC value is used to measure the informativeness of a genetic marker for linkage analysis; it was calculated according to the Botstein’s formula [26]. The genetic diversity parameters (Nei’s gene diversity GD, total genetic diversity in a population *H_T_*, and mean diversity within each subpopulation *H_S_*) were estimated according to Nei’s formula [49]. Designation GD was used to denote total genetic diversity of the whole population of 179 accessions, and total genetic diversity of the old and modern pools. Designations *H_T_* and *H_S_* were used in calculations of the basic diversity statistics for population differentiation (following Rufo et al. [33]): Jost’s index of population differentiation (*D_ST_*) [50] and coefficient of genetic differentiation (*G_ST_*) [51]. *D_ST_* and *G_ST_* were calculated using R software package *diveRsity* [52].

The Jost’s index of population differentiation (*D_ST_*) was calculated by the formula:

$$D_{ST}=\frac{H_{T}-H_{S}}{1-H_{S}}*\frac{k}{k-1}$$

where *H_T_* is the total genetic diversity in a population, *H_S_* is the mean diversity within each subpopulation, and *k* is the number of subpopulations that are sampled.

The coefficient of genetic differentiation *G_ST_* is the proportion of total genetic diversity that is distributed among subpopulations. *G_ST_* was calculated as a function of *H_T_* and *H_S_* using the formula:

$$G_{ST}=\frac{D_{ST}}{H_{T}}$$

The extent of gene flow, i.e., the transfer of genes between subpopulations was estimated by calculating the *Nm* statistic according to the formula given in [53]:

$$Nm=0.5*\frac{1-G_{ST}}{G_{ST}}$$

To estimate linkage disequilibrium (LD), the software package TASSEL v.5.0 was used [54]. LD was estimated by the square of marker correlations (*r^2^*) with a sliding window of 50 cM at a significance level of *p* < 0.001. For each genome, the critical value of *r^2^* was estimated as the mean *r^2^*. To determine how fast LD decays for each chromosome, the critical value of marker correlation (*r^2^*) was estimated as the mean *r^2^* for the chromosome, and the *r^2^* values were plotted against the genetic distance. Then, the LOESS curve [55] was fitted to determine the distance at which the curve intercepts the line of the critical value of *r^2^*. The projection of this point of intersection on the coordinate axis of the distance determines where LD becomes approximately equal to zero. The R package ggplot2 was used to plot the LOESS curves [56].

### 4.4. Population Structure

The genetic structure of the population was analyzed using the Bayesian clustering algorithm in STRUCTURE 2.3.4 software. From one to six subpopulations were suggested to analyze the Bulwheat population structure and the admixture model with 10,000 burn-in iteration, and 100,000 Monte Carlo Markov chain replications after burn-in was used [57]. The Evanno method [58] was used to calculate the number of subpopulations with STRUCTURE HARVESTER software [59]. Also, to estimate the optimal number of subpopulations, a k-means clustering algorithm from the *FactoMineR* package available in R was used. In addition, Principal Component Analysis (PCA) was applied to the data to identify and visualize subpopulations using the R software package *FactoMineR* [60]. The *factoextra* package was used to extract, visualize and interpret the results obtained by the model-based clustering algorithm and PCA.

## 5. Conclusions

The current study explored the extent of genetic diversity, linkage disequilibrium (LD) and the presence of genetic structures in a panel of 179 bread wheat old accessions and advanced cultivars collected or developed in the Eastern European country Bulgaria using SNP markers. Slight differences were noted between the old and modern collections regarding Nei’s gene diversity and PIC, and significant differences were revealed with respect to LD. The three implemented approaches—STRUCTURE, k-means clustering algorithm and PCA, partly revealed the geographical pattern of accessions distribution. The inferred structure for the modern cultivars reflected the different strategies adopted by the breeding centres. The role of the old germplasm in the contemporary breeding programs had been low. 

The detailed understanding of the genetic diversity and population structure of the Bulgarian old and modern bread wheat cultivars provided in this study could be of interest for motivating development of new cultivars with improved characteristics, especially by using the unrealized potential of the old germplasm. The obtained knowledge on SNP informativeness and population LD is a pre-requisite for further association mapping studies of important agronomic traits. 

## Figures and Tables

**Table 1:** Comparison between old and modern wheat accessions in a panel of 179 Bulgarian bread wheat accessions: SNP, SNP number; GD, Nei’s gene diversity; PIC, polymorphic information content; LD, linkage disequilibrium measures (*r^2^*, square of marker correlations, %, percentage of markers in LD at *p* < 0.001, and LD decay) for each chromosome, A, B and D genomes, and in total.

Chromosome/ Genome	Old Germplasm						Modern Cultivars					
	SNP	GD	PIC	LD (*r^2^*)	LD (%)	LD Decay	SNP	GD	PIC	LD (*r^2^*)	LD (%)	LD Decay
**1A**	1115	0.40 *	0.31 *	0.67 *	23	6	1120	0.35	0.28	0.45	30	3
**1B**	1068	0.37 *	0.29 *	0.58 *	22	3	1102	0.33	0.27	0.44	45	3
**1D**	393	0.39	0.31	0.64 *	18	3	399	0.39	0.31	0.51	31	3
**2A**	1156	0.37 *	0.30 *	0.67 *	17	2	1153	0.35	0.28	0.53	25	2
**2B**	1273	0.37 *	0.29	0.69 *	13	2	1193	0.36	0.29	0.39	32	3
**2D**	450	0.37	0.30	0.71 *	15	5	464	0.37	0.29	0.54	33	8
**3A**	1024	0.35 *	0.28	0.60 *	14	5	1064	0.36	0.28	0.41	29	4
**3B**	1264	0.37 *	0.30 *	0.56 *	23	5	1303	0.35	0.28	0.4	43	4
**3D**	241	0.38	0.30	0.71 *	9	3	243	0.37	0.29	0.49	16	3
**4A**	657	0.36 *	0.29 *	0.69 *	6	3	648	0.33	0.27	0.36	28	3
**4B**	591	0.34	0.28	0.71 *	25	6	612	0.35	0.28	0.45	39	6
**4D**	95	0.33	0.27	0.67 *	8	6	105	0.35	0.28	0.34	14	8
**5A**	1164	0.35	0.28	0.70 *	12	4	1217	0.35	0.28	0.42	37	6
**5B**	1344	0.36 *	0.29 *	0.55 *	17	3	1427	0.35	0.28	0.39	40	5
**5D**	311	0.38 *	0.30 *	0.60 *	17	2	311	0.32	0.27	0.49	23	3
**6A**	964	0.34	0.28 *	0.49 *	27	1	1093	0.34	0.27	0.44	32	2
**6B**	1093	0.38 *	0.30 *	0.68 *	13	3	1129	0.36	0.29	0.40	40	6
**6D**	303	0.36	0.29	0.71 *	13	1	308	0.35	0.28	0.56	21	3
**7A**	1275	0.36	0.29	0.71 *	11	2	1286	0.36	0.29	0.41	30	3
**7B**	918	0.36 *	0.29 *	0.67 *	13	3	933	0.35	0.28	0.36	34	4
**7D**	243	0.35	0.28	0.45 *	7	2	256	0.34	0.28	0.35	10	4
**A genome**	7355	0.36 *	0.29 *	0.65 *	19	3.3	7581	0.35	0.28	0.43	30	3.3
**B genome**	7551	0.36 *	0.29 *	0.63 *	18	3.6	7699	0.35	0.28	0.4	39	4.4
**D genome**	2036	0.37 *	0.29	0.64 *	12	3.1	2086	0.36	0.29	0.47	21	4.6
**Total/Average**	16,942	0.36 *	0.29 *	0.64 *	16	3.3	17,366	0.35	0.28	0.43	30	4.1

*- significant difference between means; α = 0.05.

**Table 2:** Distribution of 179 Bulgarian old and modern bread wheat accessions within each STRUCTURE-derived subpopulation (SP) according to the geographic region—breeding centre or collection site.

Subpopulation Region/Type	SP1		SP2		SP3		Total
	Old	Modern	Old	Modern	Old	Modern	
**Northern Bulgaria**	4	68	26	3	0	8	109
**Southern Bulgaria**	2	31	13	3	2	6	57
**Western Bulgaria**	0	4	4	0	0	0	8
**Q means**	0.719	0.707	0.830	0.632	0.739	0.721	

**Table 3:** Genetic diversity and differentiation between subpopulations (SPs) within a panel of 179 old and modern Bulgarian bread wheat accessions. SP, subpopulation; *H_T_*, total genetic diversity; *H_S_*, mean diversity within each subpopulation; *D_ST_* Jost’s index of population differentiation; *G_ST_*, coefficient of genetic differentiation; Nm, gene flow.

SP	No of Accessions	*H_T_*	*H_S_*	*D_ST_*	*G_ST_*	*Nm*
**Total**	179	0.3586	0.3481	0.0242	0.0675	6.91
**SP1**	109	0.3336	-	-	-	-
**SP2**	49	0.3419	-	-	-	-
**SP3**	16	0.3289	-			
**Admixed**	5	-	-	-	-	-
**Old**	51	0.3654	0.3419	0.0536	0.1467	2.91
**Modern**	128	0.3533	0.3313	0.0493	0.1395	3.08
**SP1-SP2**	158	0.3608	0.3376	0.0525	0.1455	2.94
**SP1-SP3**	125	0.3370	0.3313	0.0128	0.0380	12.66
**SP2-SP3**	65	0.3582	0.3353	0.0517	0.1443	2.97

## Data Availability

Not applicable.

## Supplementary Materials

The following are available online at https://www.mdpi.com/2223-7747/10/6/1116/s1, Table S1: Comparison of old and modern wheat accessions: mean, maximum, minimum, standard deviation (SD) and standard error (STD err) values for total genetic diversity *H_T_*, polymorphism information content, PIC and linkage disequilibrium, LD (*r^2^*). Table S2: List of bread wheat accessions of Bulgarian selection used in the study: Accession name, Botanical variety, Accession status, Region of breeding/collection site, Year of registration of modern cultivars, Breeding Institute, Known pedigree, Subpopulation according to STRUCTURE, Q-values, Cluster according to k-means clustering analysis.
